# Anti-ganglioside complex antibody profiles in a recurrent complicated case of GQ1b-seronegative miller fisher syndrome and Bickerstaff brainstem encephalitis: a case report

**DOI:** 10.1186/s12883-018-1077-5

**Published:** 2018-05-23

**Authors:** Hiroto Ito, Yuki Hatanaka, Yuki Fukami, Yumiko Harada, Rei Kobayashi, Hisashi Okada, Ayumi Uchibori, Atsuro Chiba, Satoshi Okuda

**Affiliations:** 1 Department of Neurology, National Hospital Organization Nagoya Medical Center, 4-1-1 Sannomaru, Naka-ku, Nagoya, Japan; 2 Department of Neurology, Kyorin University, 6-20-2 Shinkawa, Mitakai, Tokyo, Japan; 3 Department of Neurology, Teikyo University, 2-11-1 Kaga, Itabashi-ku, Tokyo, 173-8605 Japan; 4 Department of Neurology, Nagoya University Hospital, 65 Tsurumai-cho, Showa-ku, Nagoya, Aichi Japan

**Keywords:** Bickerstaff brainstem encephalitis, Miller fisher syndrome, Guillain-Barré syndrome, Anti-ganglioside complex antibodies, Recurrence, GQ1b-seronegative

## Abstract

**Background:**

Guillain-Barré syndrome (GBS), Miller Fisher syndrome (MFS) and Bickerstaff brainstem encephalitis (BBE) are a group of autoimmune neurological disorders (GBS spectrum disorder) that rarely recur. Recently, anti-ganglioside complex antibodies (GSC-Abs) were identified in patients with GBS spectrum disorder. However, there has been no case report describing GSC-Abs profiles in a recurrent case showing different phenotypes.

**Case presentation:**

We report the case of a 33-year-old male patient with GQ1b-seronegative BBE-GBS after two prior episodes of MFS-GBS. Our patient showed ophthalmoplegia, ataxia, areflexia and a weakness of the extremities (MFS and GBS symptoms) in all episodes. In the episode reported here, our patient showed disturbed consciousness and an extensor response to cutaneous plantar stimulation was observed (BBE symptoms), with severe disability and requirement for artificial respiration management. GSC-Abs detected in previous episodes were also detected in the subsequent episodes, while new GSC-Abs emerged in each episode. Interestingly, whereas antibodies to GA1/GQ1b and GA1/GT1a, which are commonly identified in patients with GBS, MFS or BBE, appeared in all episodes, antibodies to GD1a/GD1b and GD1b/GT1b, which are predominantly associated with severe disability and the requirement for artificial respiration management in GBS, emerged for the first time in this episode.

**Conclusion:**

This study reports novel phenomena about the GSC-Abs profiles and its relationship with clinical features in a case with recurrent GBS spectrum disorder, showing different phenotypes in different episodes. Further studies are required to reveal the significance of the GSC-Abs profiles in recurrent GBS spectrum disorder.

## Background

Guillain-Barré syndrome (GBS), Miller Fisher syndrome (MFS) and Bickerstaff brainstem encephalitis (BBE) are considered to form a continuous clinical spectrum, GBS spectrum disorder with monophasic symptoms [1]. When they recur, symptoms tend to be similar, although most cases exhibit some differences compared with the previous episodes [2–4]. Recently, ganglioside complexes (GSCs) such as GD1a/GD1b were identified as target antigens for serum antibodies in patients with GBS spectrum disorder [5]. However, anti-ganglioside complex antibody (GSC-Abs) profiles and their relationship with clinical features in different episodes of recurrent cases are unknown. Herein, we report the GSC-Abs profiles and clinical features of a patient with BBE-GBS but without antibodies to GQ1b after two prior episodes of MFS-GBS.

## Case presentation

**Fig. 1:**
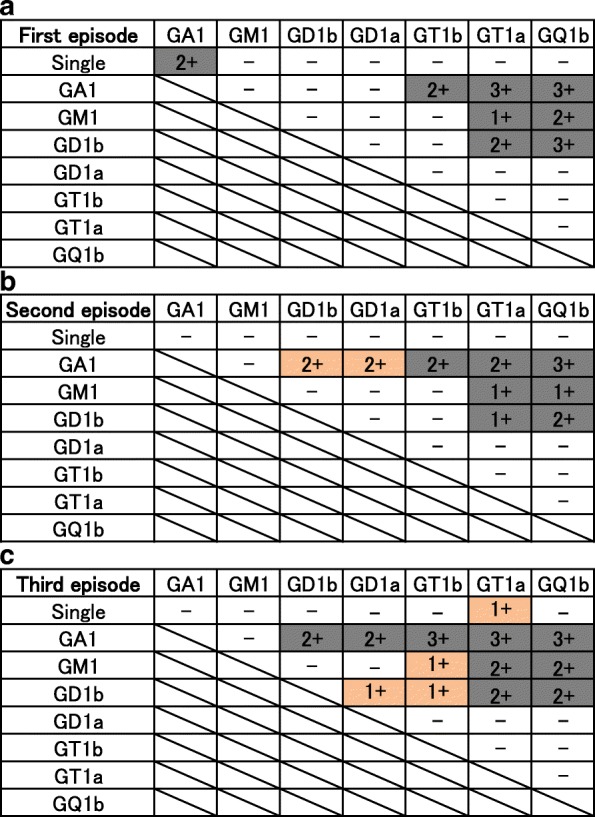
Profiles of anti-ganglioside antibodies and GCS-Abs in each episode (**a**: first episode, **b**: second episode, **c**: third episode). Isolated gangliosides and GSC were grouped into categories based on antibody titer as follows: (1+) 0.1 to < 0.5; (2+) 0.5 to < 1.0; and (3+) 1.0 or more. In all episodes, serum reacted more strongly to specific ganglioside complexes such as GA1/GQ1b and GA1/GT1a, compared with isolated gangliosides. GSC-Abs that were positive in the previous episode were also positive during the subsequent episodes, while some GSC-Abs newly appeared in each episode

A 33-year-old Japanese man presented with disturbed consciousness. He had a medical history of recurrent MFS-GBS. At age 25, in August, he developed diplopia, difficulty in speaking and swallowing, and an unsteady gait two weeks after suffering from diarrhea. On admission, he showed flaccid tetraparesis (Medical Research Council (MRC) scale grade 3 in the lower limbs, 4 in the upper limbs), external ophthalmoplegia and areflexia. His consciousness was alert, and pathological reflexes were not observed. Nerve conduction studies (NCS) on day 7 showed a slight reduction in sensory nerve action potential (SNAP, recorded orthodromically) amplitude in all examined nerves (5.5 μV in the right median nerve; normal range > 7.9 μV) and slightly prolonged distal motor latency (4.3 ms in the right median nerve only; normal range < 3.7 ms). Slightly slowed F-wave conduction velocity (FWCV) was also observed in all examined nerves (58.0 m/s in the median nerve; normal range > 59.0 m/s). Serum IgG antibodies on admission were measured by enzyme-linked immunosorbent assay to gangliosides (GM1, GM2, GM3, GD1a, GD1b, GD2, GD3, GT1a, GT1b, GQ1b, GA1) and combinations of gangliosides (GM1, GD1a, GD1b, GT1b, GQ1b, GA1). Enzyme-linked immunosorbent assay for IgG antibodies against isolated gangliosides and ganglioside complexes were performed as previously reported [6, 7]. Antibodies to GA1 and GSCs were positive, especially GA1/GQ1b and GA1/GT1a, which had high titers (Fig. 1a). After admission, the weakness in all limbs worsened, and the patient became chair-bound on day 7 after admission. He was then treated with intravenous immunoglobulin (IVIG, 0.4 g/kg/day for 5 days) and began to recover. Within a month, he was discharged with a crutch and later fully recovered. Serum reactivities to GSCs on day 30 were reduced (respective optical density (OD) values of GA1, GA1/GQ1b and GA1/GT1a were 0.69, 1.71, and 1.84 on day 1, and 0.29, 0.84 and 0.85 on day 30).

At age 28, in May, one week after suffering from a common cold, he developed an unsteady gait, diplopia and difficulty in speaking and swallowing. He was then readmitted to the same hospital. On examination, loss of proprioception was especially notable, in addition to dysarthria, ophthalmoplegia, areflexia and a weakness of the lower extremities. He was normal in mental status and did not show pathological reflexes. NCS on day 7 showed slightly prolonged distal motor latency (4.1 ms in the right median nerve only) and a slight reduction of SNAP (recorded orthodromically) amplitude in all examined nerves except for the sural nerve (4.9 μV in the right median nerve). Anti-ganglioside antibodies were negative, and antibodies to GA1/GD1b and GA1/GD1a were detected for the first time (in addition to the GSC-Abs identified in the first episode) (Fig. 1b). Starting at admission, IVIG was administered for 5 days. Although he was restricted to a wheelchair at the nadir, he left the hospital without any disability two weeks after admission.

At age 33, in July, four days after an upper respiratory infection, he noticed diplopia and weakness of the lower extremities. The following day his gait became unsteady. He then complained of a difficulty in breathing and finally displayed drowsiness, and was admitted to our hospital. On admission, he had incomplete ophthalmoplegia, slurred speech, bilateral facial weakness, ptosis and flaccid quadriparesis (MRC grade 2 in the lower limbs, 3 in the upper limbs). Intention tremor and paresthesia were observed. Deep tendon reflexes were absent and an extensor response to cutaneous plantar stimulation was observed. NCS on day 7 showed prolonged distal motor latency in almost all nerves (4.6 ms in the right median nerve) and a reduction of SNAP (recorded antidromically) amplitude in all examined nerves (1.9 μV in the right median nerve; normal range > 10.0 μV). Slowed FWCV was also observed only in tibial nerves (37.6 m/s; normal range > 45.5 m/s). Antibodies to isolated GT1a and GD1a/GD1b, GT1b/GD1b and GM1/GT1b were detected for the first time (in addition to the GSC-Abs identified in previous episodes) (Fig. 1c). His cerebrospinal fluid was abnormal, with an increase in protein (73 mg/dl) and evidence of pleocytosis (432/3 μl; polymorphonuclear/mononuclear leukocyte ratio: 45/55). Brain and spinal magnetic resonance imaging were normal. Electroencephalography on day 5 showed a slow α-rhythm and occasional diffuse theta activity. *Haemophilus influenzae* was found in his sputum culture*.*

On the day of admission, he developed a more severe disturbed consciousness, and intratracheal intubation was required for potential respiratory failure. He was given IVIG and intravenous methylprednisolone (1000 mg/day on days 1–5). On day 5, he fell into a deep coma without sedation. Starting on day 7, his condition gradually improved. First, his consciousness recovered, which revealed complete flaccid paralysis of the four limbs. Thereafter, the pathological reflexes disappeared. As his limb weakness was still severe, a second IVIG regimen was performed on days 14–18. Finally, the weakness of his four limbs gradually disappeared, and sensory ataxia became apparent. Serum reactivities to GT1a and GSCs on day 40 were reduced (respective OD values of GT1a, GA1/GQ1b and GA1/GT1a were 0.28, 1.28, and 1.29 on day 1, and 0.01, 0.24 and 0.27 on day 40). On day 56, he was transferred to a rehabilitation hospital with a wheelchair. At 10 months after the third onset, he had almost fully recovered, except for hyporeflexia and very mild diplopia and ataxia.

## Discussion

In the current case, we diagnosed the first and second episodes as MFS-GBS (first episode mainly GBS, second episode mainly MFS), and the third episode as BBE-GBS. In the third episode, in addition to the previous MFS symptoms, he exhibited new symptoms, including disturbed consciousness and extensor plantar responses, and he required artificial respiration management. Antibodies to GQ1b were negative. However, the GSC-Abs detected in previous episodes were also detected in subsequent episodes, as well as new GSC-Abs during each attack, in part reflecting his clinical features. Antibodies with high titers in the first episode, such as GA1/GQ1b and GA1/GT1a antibodies, also had high titers in recurrent episodes. These antibodies were produced and then reduced in parallel with the neurological symptoms.

The frequency of recurrence in GBS and its variants is low (reported as 2–6% in GBS) [1, 2]. In general, patients with recurrent GBS and its variants show symptoms similar to previous episodes at relapse [2–4]. The emergence of different phenotypes in subsequent episodes is rare [1–4]. In previously reported cases where different phenotypes were observed at relapse, most patients (5 out of 6 patients) developed both MFS and BBE (Table 1) [8–12], possibly because MFS and BBE have pathogenetic and clinical similarities [13]. Given that symptoms at relapse are generally similar to those at previous episodes [2–4], these reports and our case also suggest that MFS and BBE are in close proximity in GBS spectrum disorders. In all cases, BBE was followed by MFS or incomplete MFS. Three of these five patients required artificial respiration management in the last episode, suggesting disease had a tendency to be more severe in recurrent episodes, although all patients responded well to treatment and showed complete or near complete responses (Table 1).

**Table 1:** Reported cases describing the different phenotypes during various episodes

Case	Age	Sex	Episode	Preceding illness	DOC	Ophthalm oplegia	Ataxia	Areflexia	Hyper reflexia	PR	Limb weakness	GQ1b Ab	GSC Ab	Phenotype	FG at nadir	Treatment	Outcome	Reference
1	38	F	First	URI	–	+	+	+	–	–	–	+	n.e	MFS	1	IAPP	CR	[8]
	38		Second	URI	–	–	–	+	–	–	+	+	n.e	GBS	4	IAPP	CR	
2	10	F	First	URI	–	+	+	+	–	–	–	n.e	n.e	MFS	2	n.d	CR	[9]
	17		Second	n.d	–	–	+	+	–	–	–	n.e	n.e	Incomplete MFS	1	n.d	CR	
	19		Third	Fever	+	+	+	+	–	+	±	+	n.e	BBE	5	IVIG	NCR	
3	48	M	First	n.d	–	+	n.d	n.d	n.d	n.d	+	n.e	n.e	MFS-GBS	5	n.d	CR	[10]
	53		Second	–	+	+	+	+	–	+	+	–	n.e	BBE-GBS	5	IVIG	CR	
4	35	M	First	n.d	–	+	n.d	n.d	n.d	n.d	–	n.e	n.e	Incomplete MFS	1	–	CR	[11]
	38		Second	n.d	+	+	+	–	+	–	–	n.e	n.e	BBE	5	IVIG	CR	
	43		Third	Fever	+	+	+	–	+	–	–	–	n.e	BBE	5	IVIG	CR	
5	46	M	First	n.d	+	+	+	+	–	n.d	–	–	n.e	MFS (BBE)^a^	3	IVIG	NCR	[12]
	46		Second	n.d	+	+	+	+	–	+	±	–	n.e	BBE	2	IVIG	NCR	
6	25	M	First	URI	–	+	+	+	–	–	+	–	+	MFS-GBS	4	IVIG	CR	Our case
	28		Second	URI	–	+	+	+	–	–	+	–	+	MFS-GBS	4	IVIG	CR	
	33		Third	URI	+	+	+	+	–	+	+	–	+	BBE-GBS	5	IVIG	NCR	

*F* female, *M* male, *GBS* guillain-Barré syndrome, *MFS m*iller fisher syndromes, *BBE* bickerstaff brainstem encephalitis, *Incomplete FS* acute ophthalmoparesis, acute ataxic neuropathy, acute ptosis and acute mydriasis, *URI* upper respiratory infection, *DOC* disturbance of consciousness, *PR* pathological reflex, *Limb weakness +* MMT ≤ 3 at least one limb, *Limb weakness ±* MMT ≥ 4, *MFS-GBS* MFS with definite limb weakness (MMT ≤ 3), *BBE-GBS* BBE with definite limb weakness (MMT ≤ 3), *n.e* not examined, *n.d* not documented, *FG* fuctional grade, *IAPP* immunoadsorption plasmapheresis, *IVIG* intravenous immunoglobulin, *CR* complete recovery, *NCR* near complete recovery (Mild diplopia, weakness, and atxia without disability)

^a^Although this case was considered “Fisher-Bickerstaff syndrome” and the first episode was considered MFS in reference [9], we consider the first episode as BBE because of the presence of drowsiness [1]

The continued presence of some GSC-Abs and the emergence of new antibodies in our case are consistent with the generally similar, but at the same time unique, clinical features. Our patient exhibited ophthalmoplegia, ataxia, areflexia and a weakness of the extremities in each episode. Antibodies to GA1/GT1a and GA1/GQ1b (commonly identified in patients with GBS, MFS or BBE), GM1/GQ1b, and GD1b/GT1a (commonly identified in patients with GBS or MFS) appeared in all episodes [5]. Antibodies to GD1a/GD1b and GD1b/GT1b, which emerged only in the third episode, are predominantly associated with severe disability in GBS and the requirement for artificial respiration management [5]. Recently, complex enhanced or attenuated anti-GQ1b or GT1a antibodies were reported to underlie the different clinical features in anti-GQ1b antibody syndrome [14]. In our case, subtle differences in the specificities of GSC-Abs in each episode may account for the differential clinical features observed.

The profiles of antibodies to isolated gangliosides and GSCs may be related to recurrence. Previous studies revealed a high frequency of IgG antibodies to GQ1b in MFS patients (83–95%) [6, 13, 15] and BBE patients (66–68%) [13, 16]. By comparison, GQ1b-seronegative and GSC-seropositive MFS patients represented only 4 (1.9%) of 207 MFS patients [15]. Interestingly, those patients were all male, and one of them relapsed [15], similar to our case. The relapsed patient in that study had antibodies to GM1/GQ1b and GM1/GT1a in the second episode [15], which also continued to be positive in our case. Furthermore, we reviewed the positive rate of antibodies to GQ1b in reported recurrent cases where patients showed both BBE and MFS. During recurrence, among patients who showed both BBE and MFS, only 1 female patient out of 5 (20.0%) was positive for antibodies to GQ1b, and the other GQ1b-seronegative patients were male (Table 1) [8–12]. This rate for antibodies to GQ1b was low compared with 83–95% in patients with monophasic MFS and 66–68% in patients with monophasic BBE [6, 13, 15, 16]. Some of the patients with both BBE and MFS may have had GQ1b-seronegative and GSC-seropositive profiles.

## Conclusion

We observed novel phenomena in this recurrent complicated case of GQ1b-seronegative MFS-GBS and BBE-GBS. GSC-Abs that were positive in previous episodes were also detected in subsequent episodes, while new GSC-Abs appeared in each episode, partly reflecting the clinical features. Based on our review of previous reports, GQ1b-seronegative and GSC-seropositive profiles in patients with MFS or BBE might be associated with recurrence, but further studies are needed to identify patients with a risk for recurrence.

## Abbreviations

BBE: Bickerstaff brainstem encephalitis
FWCV: F-wave conduction velocity
GBS: Guillain-Barré syndrome
GSC-Abs: Anti-ganglioside complex antibodies
IVIG: Intravenous immunoglobulin
MFS: Miller Fisher syndrome
MRC: Medical Research Council
NCS: Nerve conduction studies
OD: Optical density
SNAP: Sensory nerve action potential
